# Wide-Angle Scanning Graphene-Biased Terahertz Coding Meta-Surface

**DOI:** 10.3390/mi14020233

**Published:** 2023-01-17

**Authors:** Yangyang Xu, Rui Yang, Yan Wang

**Affiliations:** 1 National Key Laboratory of Antennas and Microwave Technology, School of Electronic Engineering, Xidian University, Xi’an 710071, China; 2 Beijing Institute of Astronautical System Engineering, Beijing 100076, China

**Keywords:** coding meta-surface, graphene, wide-angle scanning, frequency scanning

## Abstract

We demonstrate a reconfigurable beam steerable meta-surface through a graphene-biased slot-array over a grounded quartz substrate. More specifically, the graphene meta-elements can be dynamically tuned to program the radiations by applying adequate DC bias voltages to different gating pads, capable of turning on or off the releasing slots of the guided fields as adjustable switches. In particular, such a graphene-biased terahertz meta-surface will achieve a wide-angle steerable beam at a fixed frequency and the scanning directions can further be modulated when varying the frequency at a certain state of the graphene, thus should pave the way for building up more advanced reconfigurable transceivers and sensors in terahertz wireless electronics.

## 1. Introduction

Graphene is a two-dimensional material composed of carbon atoms arranged in a hexagonal lattice. The extraordinary electronic, optical, and thermal characteristics of graphene as well as the possibility of facile integration have enabled the great applications of terahertz graphene meta-surfaces. In particular, graphene meta-surfaces have been widely applied to the reconfigurable electronics [1] and proved to be valid candidates for the synthesis of beam forming due to the increasing availability of synthesized graphene through micromechanical exfoliation, growth on various substrate, and deposition. For example, Zhaoyi Li et al. theoretically demonstrated the modulation of phases with graphene by tuning the resonance of a plasmonic meta-surface [2]. The graphene-based reflectarray was presented by Carrasco et al., where the tunability of reflected radiation was dependent on the chemical potential induced phase variations [3]. Zubin Li et al. proposed graphene ribbon meta-surfaces achieving the anomalous reflection and the dynamical tuning of non-diffractive beams [4]. Deng et al. designed a wide-band and high-gain circularly polarized graphene reflectarray in the THz regime with Pancharatnam–Berry phase scheme [5]. Chang et al. proposed a reconfigurable graphene reflectarray for the generation of vortex waves at THz with a normal incident plane wave [6]. Meng et al. proposed a graphene transmit-array to generate the tunable vortex wave in a wide band with multilayer metamaterial elements [7]. These works, fundamentally dependent on the gradient phase distributions of meta-surfaces by adjusting the graphene chemical potential or shape continuously, have all fulfilled the desired regulations of electromagnetic fields.

On the other hand, coding meta-surfaces with dual states of “0” and “1” can sufficiently control the scattering characteristics, where PIN-diodes or varactors integrated with meta-units are usually employed to build up the reconfigurable meta-arrays mostly in the microwave regime. For example, Cui et al. initially proposed the concept of digital coding and programmable meta-surface composed of two types of units, with 0 and π phase responses. By coding these units, one can control the radiation beams of antennas and reducing the scattering features of targets [8]. Li et al. employed the reprogrammable holograms to generate various high-resolution and low-noise holographic images by providing switchable phase modulations at microwave frequency [9]. Tao et al. demonstrated a single reconfigurable device using PIN-diodes to control polarization of waves and transform the state of waves between transmission and reflection [10]. Yurduseven et al. presented a reconfigurable holographic meta-surface to tune near-field beam-focusing in the Fresnel zone by switching PIN-diodes [11] and also proposed a single-frequency all-electronic microwave camera at K-band frequency by a reconfigurable and dynamic beam-steering holographic meta-surface aperture [12]. Li et al. presented a novel SIW programmable meta-surface to manipulate electromagnetic waves in real-time and generate narrow scanning beams with wide scanning angles [13]. However, such electrical circuits are not applied in the terahertz region due to size issues and high coupling loss. Graphene is ready to fill such a vacancy on the merits of one-atomic thickness and the substantial characteristic of electromagnetic field-induced tunability [14,15]. Graphene is also able to function as adjustable switch at terahertz by simply applying adequate DC bias voltages, thus should initiate the quest for tangible applications in controlling electromagnetic fields with much more freedom [16,17]. Based on these considerations, we demonstrate a reconfigurable beam steerable meta-surface through a graphene-biased slot-array over a grounded quartz substrate. Each releasing slot can be modeled as a polarizable dipole transforming guided modes to the radiations [18]. Especially, the coding characteristic of graphene meta-elements can be dynamically tuned to program the radiations to different gating pads and turn on or off the releasing slots of the guided fields. We show that such a graphene-biased terahertz meta-surface will achieve a wide-angle steerable beam at a fixed frequency and the scanning directions can further be modulated when varying the frequency at a certain state of the graphene.

## 2. Design and Results

**Figure 1:**
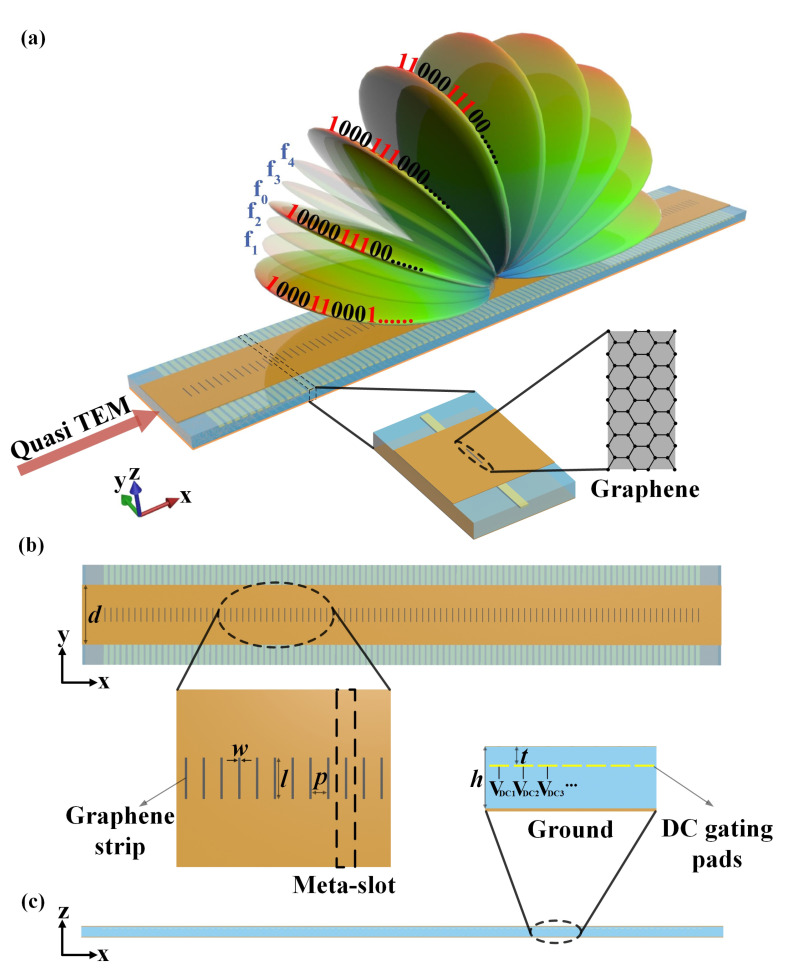
Schematic view of the graphene-biased terahertz coding meta-surface for the synthesis of scanning beams. (**a**) Full view. (**b**) Top view. (**c**) Cross-sectional view along the *x*-direction.

Figure 1 demonstrates the configuration of the graphene-biased terahertz coding meta-surface and the synthesis of the desired scanning radiations. It consists of a series graphene strips in the gap of the slot array over a grounded quartz substrate ($\varepsilon_{r}=3.8$), with the polysilicon pads to modify the graphene surface conductivity as a function of the applied DC voltage. The polysilicon pads can be safely neglected because they are thin and with a relative permittivity ($\varepsilon_{r}=3$) similar to the quartz substrate [19]. Considering the practical implementation method, the polysilicon pads layer is deposited on the substrate, then an insulating layer is added above it. Graphene is grown using chemical vapor deposition (CVD) on Cu catalyst, then subsequently transferred on the insulating layer and patterned through electron-beam lithography [20]. The proposed graphene meta-surface with desired ability can be realized through such a growth and transfer method. By adjusting the chemical potential of the graphene meta-element in the slot, the radiation state of each meta-slot will be switched to prescribe the desired radiation, thus should fulfill the perfect coding functionality of the beam-steerable meta-surface. The meta-surface consists of 100 meta-slots, and the structural parameters of the graphene-biased terahertz coding meta-surface are l=35μm, w=2μm, p=15μm, h=25μm, t=20 nm, and d=150μm.

The surface conductivity of graphene in the low terahertz band can be described by the Kubo formalism [21],

(1) $$\sigma =-j\frac{{q_{e}}^{2}k_{B}T}{\pi \hbar^{2}\left(\omega -j2\Gamma \right)}\left[\frac{\mu_{c}}{k_{B}T}+2\ln\left(e^{-\frac{\mu_{c}}{k_{B}T}}+1\right)\right],$$

where $q_{e}$ is electronic charge, $k_{B}$ is Boltzmann’s constant, *ℏ* is the reduced Planck constant, $\mu_{c}$ is the graphene chemical potential with electrically tunable characteristics, ω is the frequency. In this paper, the relaxation time is τ=1 ps, the room temperature is T=300 K. According to the Kubo formula, the simulated graphene layer is usually regarded as an impedance surface or ultra-thin bulk layer. The conductivity of graphene can be tuned by manipulating its chemical potential via polysilicon pads applying DC voltage. This feature enables us to design graphene surface conductivity, and then manipulate the graphene plasmon to achieve the tunable electromagnetic response of the graphene-biased coding meta-surface. For non-chemically doped graphene, the carrier density $n_{s}$ of graphene can be adjusted by the bias voltage $V_{DC}$:

(2) $$n_{s}=\frac{V_{DC}\varepsilon_{r}\varepsilon_{0}}{q_{e}t}.$$

The chemical potential $\mu_{c}$ is related to the carrier density $n_{s}$,

(3) $$n_{s}=\frac{2}{\pi \hbar^{2}v_{f}^{2}}\int_{0}^{\infty }\varsigma \left[f_{d}\left(\varsigma \right)-f_{d}\left(\varsigma +2\mu_{c}\right)\right]d\varsigma ,$$

where $v_{f}\approx 9.5\times 10^{5}\;m/s$ is the Fermi velocity, $f_{d}\left(\varsigma \right)={\left(e^{\left(\varsigma -\mu_{c}\right)/k_{B}T}+1\right)}^{-1}$ is the Fermi–Dirac distribution. In order to create different radiation modes, the bias voltage imposed on each gating pad can be adjusted independently. The chemical potential 0 eV enables the graphene-biased meta-slot to function as the on-state of “1” corresponding to 0V DC voltage, while the chemical potential 1 eV enables the graphene-biased meta-slot to perform as the off-state of “0” corresponding to 75V DC voltage.

**Figure 2:**
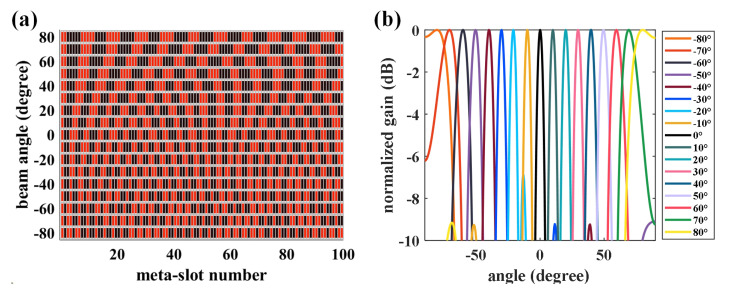
Beam-steering characteristics of the graphene-biased terahertz coding meta-surface. (**a**) 1/0-states of per element. (**b**) Scanning beams.

The far-field radiation pattern of the graphene-biased terahertz meta-surface can be approximated by accumulating the fields released from all the meta-slots [22]:

(4) $$AF=\sum_{i=1}^{N}\alpha_{m,i}e^{-j\beta x_{i}}e^{jkx_{i}\sin\theta },$$

where $\alpha_{m,i}$ is the effective magnetic polarizability. β is the waveguide constant and $x_{i}$ is the reference position from the origin. We assume that the elements are non-interacting to simplify the theoretical analysis. Given the desired radiating direction $\theta_{0}$, the required field distribution $P=e^{-jkx_{i}\sin\theta_{0}}$ over the meta-surface aperture can be obtained as the transformation of the guided wave $H=e^{-j\beta x_{i}}$ by implementing the amplitude modulation and $\alpha_{m,i}$ can be simplified as

(5) $$\alpha_{m,i}=X_{i}+M_{i}\Theta \left\{\cos\left[\left(\beta -k\sin\theta_{0}\right)x_{{}_{i}}\right]\right\},$$

with $\Theta \left(x\right)$ functioning as a step function. $X_{i}$ and $M_{i}$ are the positive real numbers to adjust the distribution range of amplitude values, and we have $X_{i}=1,\;M_{i}=0.85$ here corresponding to the energy radiated from per meta-slot. When the phase difference $\Delta =\left(\beta -k\sin\theta_{0}\right)x_{{}_{i}}$ between the required phase shift value $-kx_{i}\sin\theta_{0}$ and the actual phase shift value $-\beta x_{i}$ at position $x_{i}$ is small, more energy will be radiated as the state “1”. On the other hand, when Δ is large, less energy will be radiated as the state “0”. On this basis, the meta-slots possessing the phase difference within $-\frac{\pi }{2}+2n\pi \leq \Delta \leq \frac{\pi }{2}+2n\pi \;\left(n=0,\pm 1,\pm 2,\ldots \right)$ will significantly contribute to the overall radiation, while the rest of the meta-slots will thus exhibit less contribution to the radiation. The ideal relationship of the 1/0-state of meta-slots over the entire radiating aperture and the prescribed radiating directions can thus be determined, as shown in Figure 2a, with the red ribbon indicating the state “1” and the black ribbon indicating the state “0”, where the graphene-biased terahertz meta-surface has 100 switchable meta-slots. The very closely packed meta-slots fulfill the wide-angle scanning capacity, and we can observe that the ideal steerable beams possess the scanning range from $-80^{\circ }$ to $80^{\circ }$, as shown in Figure 2b. Unlike the phase array and electronic scanning antenna structure, the amplitude modulation does not need active phase shifters to achieve reconfigurable operations. The meta-slots are equivalent to magnetic dipole coupling guided-wave energy to the free space, and all the meta-slots thus finally form the radiating array. In fact, such an amplitude modulation also includes the phase information, where the guided wave controls the phase of each unit, and we only let the elements meet the phase requirements to radiate.

**Figure 3:**
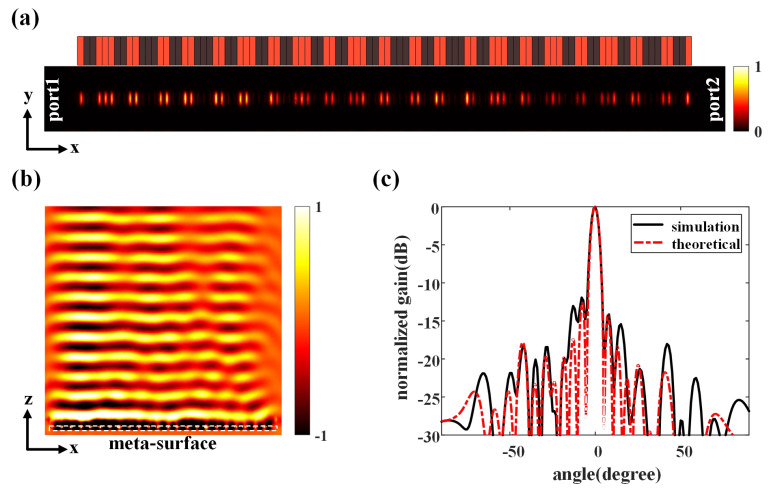
Full wave simulation of the graphene-biased terahertz coding meta-surface at 2.2 THz. (**a**) E-field distributes on the structure of the meta-slot array. The normalized radiating amplitude of the meta-slot is the *x*-component of electric field at 2.5μm above the center of each meta-slot. (**b**) Electric field leakage from the proposed design. (**c**) Normalized radiation patterns.

Full-wave simulations (CST Microwave Studio) are carried out in Figure 3 to verify the proposed graphene-biased terahertz coding meta-surface. The elements in different working states will have different effects on guided wave, as the radiation from the meta-slots will affect the transmission of the guided wave and lead to the energy attenuation of the guided wave with additional phase shift [23]. When a low bias voltage is applied to the graphene through gating pads with the chemical potential of 0eV, the meta-slot will function as dielectric windows with very good transmissivity. In such a case, the meta-slot can be modeled as a polarized magnetic dipole with strong radiation ability. The coding graphene element imposed with 0 eV chemical potential will thus turn on the meta-slot as the state “1” for the radiation, and each “1” state of the graphene element introduces a 3.5-degree phase shift to the guided wave. On the other hand, when the chemical potential of the graphene is 1 eV by applying a higher bias voltage to the graphene, the transmissivity of the meta-slot will become very small, and its physical properties will be closer to the conductor. At this time, the meta-slot is similar to the electromagnetic shielding screen with extremely weak radiation capacity of the leakage fields. The influence of such graphene elements on the guided wave can be ignored. Considering the additional phase shift introduced by the element to the guided wave, we correct the phase difference $\Delta =\left(\beta -k\sin\theta_{0}\right)x_{{}_{i}}+\delta$, where δ indicates that each 1-state element will accumulate a corresponding phase shift to the guided wave. Figure 3a shows the 0/1-state distribution which is slightly different from the ideal state distribution after introducing phase shift and the normalized electric field amplitude distribution at 2.5μm above the structure of the meta-slot array for 0-degree radiating direction, where port-1 is excited by the quasi-TEM mode and port-2 at the end of the waveguide is used to absorb the remaining energy. Figure 3b shows the normalized electric field magnitude over the transversal plane of the proposed meta-surface. The energy gradually radiates into the free space when the quasi-TEM wave travels along the structure. The electric field intensity distribution obtained by simulation is consistent with the expected results. We can observe that the proposed graphene-biased terahertz coding meta-surface forms an electric field distribution similar to a plane wave propagating in the *z*-direction which generates a highly directive beam as the numerical analysis. The HPBW of the simulated meta-surface is $5^{\circ }$, and the first sidelobe level is −11.4 dB with the beam pointing at $\theta_{0}=0^{\circ }$, exhibiting good agreement with the theoretical result, as shown in Figure 3c. The gain of the simulated result is reported to be 11 dBi with 16% total efficiency. The efficiency can be further improved by optimizing the phase threshold and using other modulation schemes, such as grayscale modulation. According to the electric field direction in the meta-slot, the far field is *x*-polarized and the cross polarization ratio is above 30 dB. The total energy provided in the simulation is 0.5 W, the energy lost in graphene is 0.31 W, and the radiation energy accounts for 0.08 W. The rest of the energy is reflected at the input port or absorbed by the terminal matching load. The slight discrepancy of far field patterns between the theoretical and simulated results is mainly attributed to the coupling between meta-slots.

**Figure 4:**
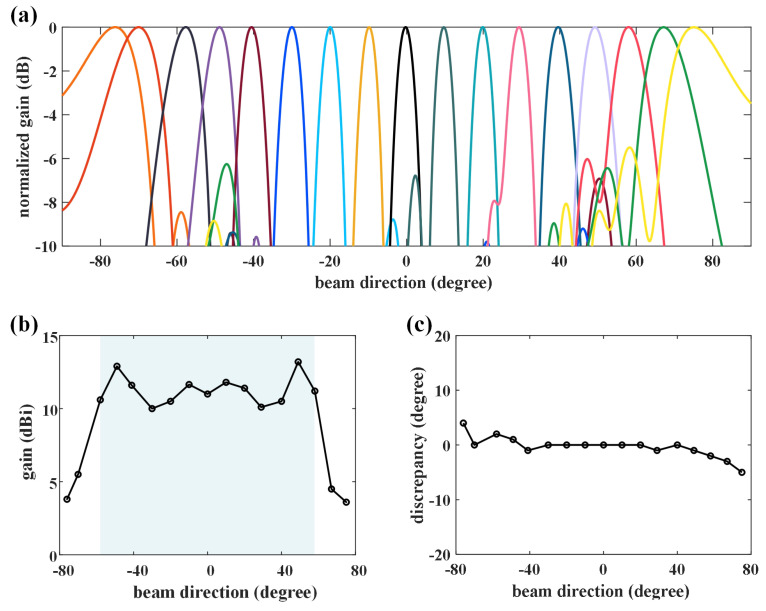
Beam-steerable characteristic of the graphene-biased terahertz coding meta-surface at 2.2 THz. (**a**) Radiation patterns at different scanning angle. Different colors represent different beam directions. (**b**) The gains of each beam at different radiating directions. (**c**) The discrepancy of the beam directions between the theoretical calculation and the simulation.

Figure 4 thus demonstrates the beam-steering performance of the proposed graphene-biased terahertz coding meta-surface. Figure 4a demonstrates the wide angle scanning of the proposed graphene-biased terahertz coding meta-surface, where 17 beams are selected to cover the scanning range of $151^{\circ }$ from $-76^{\circ }$ to $75^{\circ }$. The gain values in the main beam directions of all the patterns are shown in Figure 4b, and they are higher than 10 dBi within the scanning range of $\left[-58^{\circ },58^{\circ }\right]$. When the scanning angle gets larger, the gain will drop accordingly due to the reduction of the effective aperture. The discrepancies between the simulation and the theoretical results are less than 5 degrees, as shown in Figure 4c. It is noted that in such a simulation, we use the ideal monolayer graphene model without considering the defects produced in the practical processing of graphene. With a predetermined bias voltage, the non-perfectness of graphene may make the chemical potential no longer 0 or 1 eV. This will lead to a deviation between the actual beam direction and the expected direction. When the chemical potential changes in a small range, the directional beam can still be formed, but the beam direction will change. At the same time, the realized gain will decrease. The variation of chemical potential has a significant impact on the radiation pattern, because the influence of chemical potential on graphene is not linear.

**Figure 5:**
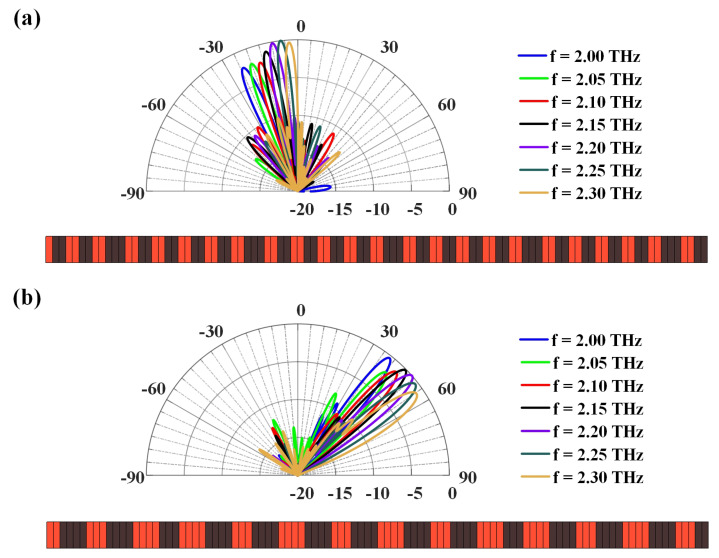
Frequency scanning of the graphene-biased terahertz coding meta-surface from 2 THz to 2.3 THz. Scanning beams of the meta-slot array at a certain coding state with the original radiation of $\theta_{0}\;=\;-10^{\circ }$ (**a**) and $\theta_{0}\;=\;50^{\circ }$ (**b**).

Figure 5 continues to demonstrate the frequency scanning capacity of the proposed graphene-biased terahertz coding meta-surface, where the scanning directions can further be modulated when varying the frequency without changing the coding state of the meta-slot array. For example, the scanning range will have $21^{\circ }$ for the original $-10^{\circ }$ scanning beam when the operating frequency varies from 2 THz to 2.3 THz, as shown in Figure 5a. A lower frequency will have a bigger scanning angle in the backward scanning range, and a higher frequency will make the radiating direction move forward. Every 0.05 THz variation in the operating frequency will lead to an average $3.5^{\circ }$ steerable beam. Similar results can also be obtained for the original $50^{\circ }$ scanning beam when the operating frequency varies the same, as shown in Figure 5b. In such a case, a higher frequency will achieve a larger scanning angle and possess a bigger forward radiating direction, while the lower frequency will have a smaller scanning angle, with every 0.05 THz variation in the operating frequency resulting in a $3^{\circ }$ steerable beam. The 0/1-state distributions for the original radiation of $\theta_{0}=-10^{\circ }$ and $\theta_{0}=50^{\circ }$ are also included. Compared with changing the coding state to achieve wide-angle scanning, the frequency-scanning angle range under a fixed state is smaller, but continuous beam scanning will be achieved by combining the two modulation schemes.

## 3. Conclusions

In conclusion, we have demonstrated a wide-angle scanning graphene-biased terahertz coding meta-surface in this paper. Especially, the proposed graphene-biased terahertz meta-surface has achieved a wide-angle steerable beam at a fixed frequency and the scanning directions can further be modulated when varying the frequency at a certain coding state of the meta-slot array. Excellent agreement is obtained between the theoretical results and simulated results. The graphene meta-elements can be dynamically tuned to program the radiations by applying adequate DC bias voltages to different gating pads, capable of turning on or off the releasing slots of the guided fields as adjustable switches. Such an amplitude modulation process does not require active phase shifters, but can also include the phase information, where the guided wave controls the phase of each unit. We expect the design strategy of using the graphene as adjustable switches to build up a programmable beam scanner in terahertz wireless electronics, paving the way for the quest of tangible applications and more advanced meta-devices in controlling the electromagnetic field with more freedom.

## Data Availability

The data presented in this study are available from the corresponding author upon reasonable request.
